# Primary sarcoma of the heart: case report and literature review

**DOI:** 10.1186/s13019-020-01157-4

**Published:** 2020-05-19

**Authors:** Rieneke Moeri-Schimmel, Elisabeth Pras, Ingrid Desar, Stijn Krol, Pètra Braam

**Affiliations:** 1 Department of Radiation Oncology, Leiden University Medical Center, Leiden, The Netherlands; 2 Department of Radiation Oncology, University Medical Center Groningen, Groningen, The Netherlands; 3 Department of Medical Oncology, Radboud University Medical Center, Nijmegen, The Netherlands; 4 Department of Radiation Oncology, Radboud University Medical Center, Geert Grooteplein Zuid 32, 6525 GA Nijmegen, The Netherlands

**Keywords:** Cardiac sarcoma, Sarcoma of the heart, Radiotherapy, Angiosarcoma, Intimal sarcoma

## Abstract

**Background:**

Primary cardiac tumors are extremely rare. Most primary cardiac tumors are benign and around one quarter is malign. Sarcomas are accounting for 95% of these malign tumors and they show different histologies. The prognosis is poor with a mean survival of 3 months to 1 year, even with complete radical resection. We report the cases of two patients with primary cardiac sarcoma treated with surgery and radiation and/or chemotherapy. In addition we retrospectively collected data of patients with primary cardiac sarcoma treated between 2005 and 2019 with minimum follow-up of 12 months. Clinical characteristics, treatment modalities and outcomes were collected and analyzed. Finally a literature review was done.

**Case presentation:**

The first patient presented with cerebellar infarction. When she developed a recurrence analysis showed a suspicious myocardial lesion for which irradical surgery (R2) was performed. Histopathology showed an intimal sarcoma of the left atrium. Postoperative radiotherapy was applied without complications. Three months after treatment multiple metastases were diagnosed and she died 13 months after initial diagnosis. The second patient presented with pericardial effusion. A tumor was found located in the right atrium and radical surgery was performed. Histopathology showed an angiosarcoma, without signs of metastases. Adjuvant radiotherapy was added because of close margins and based on high risk of recurrence and metastases it was decided to add chemotherapy. One year after finishing treatment, evaluation showed local recurrence together with pulmonary metastases.

**Conclusions:**

Surgery combined with postoperative radiotherapy is feasible in patients with resectable cardiac sarcoma. Distant metastases occur frequently. In patients with an irresectable sarcoma of the heart primary radiotherapy should be considered.

## Background

Primary cardiac tumors are extremely rare, with an autopsy incidence ranging from 0,001% to 0,030%. Most primary cardiac tumors are benign and around one quarter is malign. Sarcomas are accounting for 95% of these malign tumors and they show different histologies. Angiosarcomas, undifferentiated sarcomas and undifferentiated pleomorphic sarcoma are the most common types with a frequency of respectively 37, 24% and 11–24% [1]. Less frequent histology shows leiomyosarcoma, osteosarcoma, rhabdomyosarcoma, fibrosarcoma or synovial sarcoma. The least reported primary cardiac tumor is intimal sarcoma [2]. Primary cardiac sarcomas mainly affect adults with a mean age of 41 years at presentation [3].

Optimal treatment is to obtain a complete surgical resection [1, 4], but this is possible in less than 50% of patients [2]. The prognosis is very poor with a mean survival of 3 months to 1 year [1], due to diagnostic delay, therapeutic difficulty and high metastatic potential [5]. For patients who underwent complete surgical resection, life expectancy is twice as long as for patients who underwent an incomplete surgical resection [6, 7]. Local recurrence and metastases occur frequently and usually within 1 year [2, 3]. This report presents two cases with primary cardiac sarcoma. Besides a retrospective analyses was performed on all patients diagnosed with cardiac sarcoma in three dedicated sarcoma centers in the Netherlands, which were treated by surgery with adjuvant radiotherapy / chemotherapy or radiotherapy as primary treatment. In addition a comprehensive review of the literature is provided.

## Case presentation

### Case 1

**Fig. 1:**
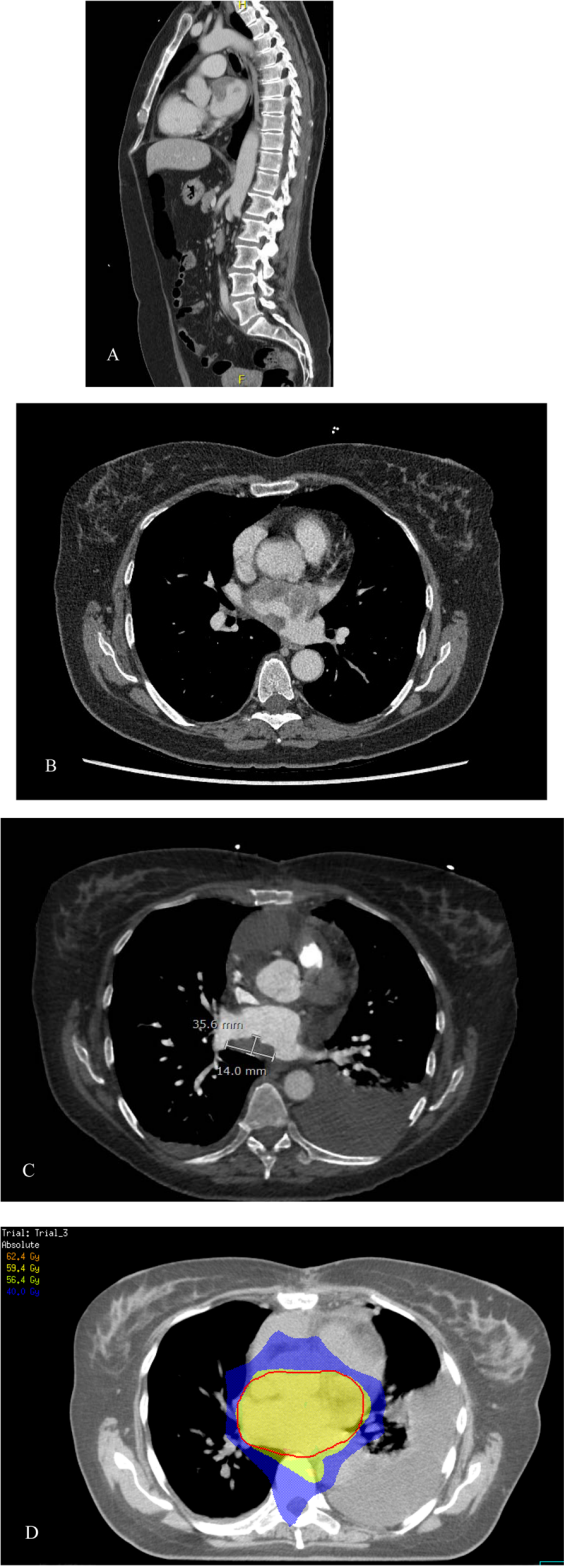
**a**. CT shows a mass located at the ventral and cranial side of the left atrium expanding into the dorsal site, in sagittal view. **b**. Transversal view. **c**. CT made 3 weeks after surgery shows remaining tumor. **d**. Radiotheraphy planning in transversal view. The red colored line reperesents the planning traget volume

A 58 year old woman presented with cerebellar infarction in the beginning of 2017. Additional investigations did not show a source for thromboembolisms. Nevertheless, at the end of the year she had a recurrence. Analysis now showed a suspicious myocardial lesion (Fig. 1) for which irradical surgery (R2) was performed. Histopathology showed an intimal sarcoma of the left atrium. Postoperative radiotherapy was applied until a total dose of 59.4 Gy in 33 fractions of 1.8 Gy using Volumetric Modulated Arc Therapy (VMAT). No complications occurred during treatment. Three months after finishing radiotherapy a PET-CT showed multiple metastases. Palliative treatment with pazopanib 800 mg OD was started, but evaluation after 2 months showed progressive disease and the treatment was stopped. She died 13 months after initial diagnosis.

### Case 2

**Fig. 2:**
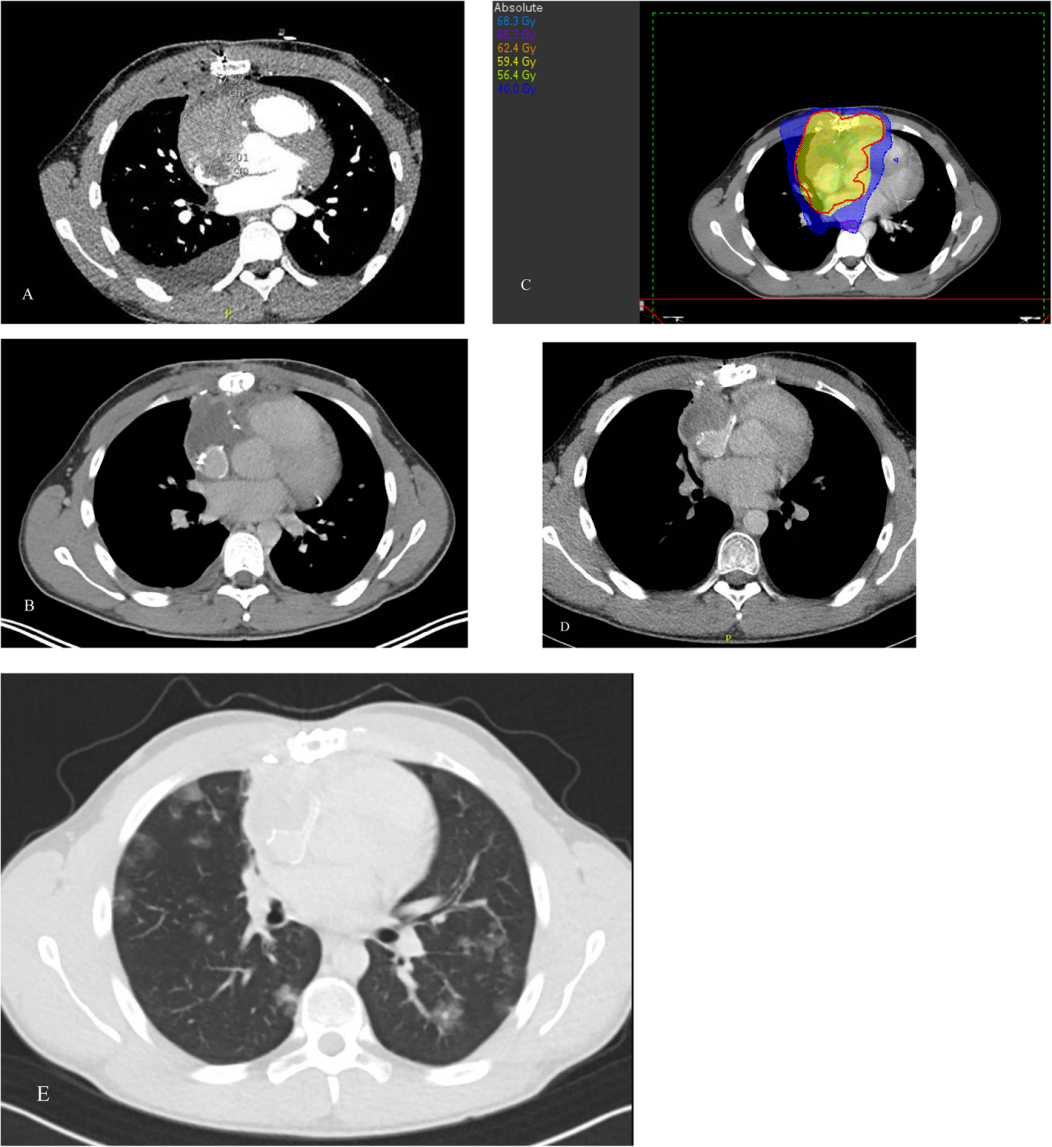
**a**. CT shows an inhomogenous mass located in the right atrium, expanding towards the pericardium and the anterior Mediastrium, with ingrowth into the superior vena cava. **b**. Scan made 3 weeks after surgery. **c** Radiotheraphy planning in transversal view. The red colored line represents the planning target volume. **d**. No evidence of disease 1 year after diagnosis. E. One year after finishing treatment CT shows local recurrence and mestastasis

A 20 years old male presented in February 2017 with a pericardial effusion. Cytology showed no signs of malignancy and at follow-up 2 months later the pericardial effusion was resolved spontaneously. Unfortunately, it recurred in December 2017. At this time, a tumor was found in the right atrium (Fig. 2) and radical surgery (R0) was performed. Histopathology showed an angiosarcoma. No metastases were found. Adjuvant radiotherapy (33 × 1.8 Gy) was added until March 2018, without complications. Based on the young age and high risk of recurrence and metastases, it was decided to add 6 cycles of paclitaxel 80 mg/m2 days 1–8-15 q28 days. One year after, evaluation showed local recurrence together with pulmonary metastases. The patient is still alive.

## Discussion and conclusions

Primary cardiac tumors are extremely rare [1]. When malign, sarcomas make up for the majority with a heterogeneous group of histologies. Information from literature about cardiac sarcomas is scarce and mainly comprises single patient case reports.

A retrospective analysis of all patients with primary sarcoma of the heart treated with curative intent in a dedicated sarcoma expertise center in the Netherlands (Leiden University Medical Center, University Medical Center Groningen, Radboud University Medical Center Nijmegen) revealed 8 patients, including the two case presentations. All patients had been diagnosed between 2005 and 2019 with at least 12 months follow-up. Clinical characteristics, treatment modalities and outcomes were collected and analyzed. Patient characteristics are presented in Table 1. In 5 patients, the tumor was located in the left atrium, in 2 patients in the right atrium. In one patient, the specific location, apart from being located in the heart near the pulmonary trunk, was not further specified. The clinical presentation was pericardial effusion in 2 patients, diagnostic suspicion of myxoma in 2 patients, 1 patient experienced cerebellar infarction, 1 patient had a collapse and in 3 patients the presentation is unknown. Four patients had cardiac angiosarcomas, 3 intimal sarcomas and one synovial sarcoma.

**Table 1:** Clinical data

Case	Age (y)/ sex	Histology	Treatment	RT dose (Gy)/fractions	Rec/met (mo)	Follow-up (mo)
1	58/F	Intimal sarcoma	R2, adj RT	59.4/33	- / + (3)	Died (13)
2	20/M	Angiosarcoma	R0, adj RT, CT	59.4/33	+ / + (20)	Alive (20)
3	34/M	Intimal sarcoma	Rx, adj RT	66.0/37	- / + (x)	Died (84)
4	60/F	Angiosarcoma	RT	60.0/30	- / + (12)	Died (24)
5	65/F	Synovial sarcoma	Rx, adj RT	59.4/33	+ / + (12)	Died (24)
6	57/M	Intimal sarcoma	R0, adj RT	59.4/33	+ / + (35)	Died (45)
7	61/F	Angiosarcoma	R1, adj RT	54.0/30	- / -	NED (12)
8	17/M	Angiosarcoma	R0, CT^a^	60.0/30^b^	+ / - (48)	NED (86)

*F* female, *M* male, *y* years, *R* resection status, *adj* adjuvant, *RT* radiotherapy, *CT* chemotherapy, *Rec/met* recurrence/metastases, *mo* months, *NED* no evidence of disease

^a^ adjuvant adriamycin and ifosfamide

^b^ patient developed a local recurrence 4 years after initial diagnosis which was treated by resection and adjuvant radiotherapy

One patient had irresectable disease and was treated with primary radiotherapy. Seven tumors were treated by resection. R0 status was reached in three patients. All other cases were documented with R1 (*n* = 1), R2 (*n* = 1) or unknown (*n* = 2). Six patients received adjuvant radiotherapy with doses between 54-66Gy. The seventh patient received adjuvant chemotherapy. No patient experienced grade ≥ 3 adverse events on either radiotherapy or chemotherapy. Documented radiation toxicity were fatigue (*n* = 1) and dizziness (n = 1).

Local recurrence without distant metastases occurred in the patient treated with adjuvant chemotherapy after primary presentation, 48 months after initial diagnosis. R1 surgery was performed followed by adjuvant radiotherapy (60Gy in 30 fractions). This patient is still alive more than 8 years after initial diagnosis (case 8). Three patients developed a local recurrence together with distant metastases, three patients developed distant metastases without signs of local recurrence. Median time between initial diagnosis and local recurrence and/or metastasis was 16 months with a range between 3 and 48 months. Median survival of these patients was 24 months (range 12–84 months) as from diagnosis. Three patients are alive after initial diagnosis with follow-up range from 12 to 86 months.

Primary cardiac tumors can arise in any part of the heart and symptoms depend on the location and on the extent of the tumor [1]. The recognition of a primary cardiac sarcoma can be difficult, as was seen in our patients 1, 5 and 7. Dyspnea is the most frequent symptom, followed by chest pain, congestive heart failure and palpitations [7]. For diagnosis echocardiography, preferably transesophageal, is a reliable and an inexpensive way to show tumor size, location, mobility and attachment [7–9]. MRI, CT and PET/CT are complementary to achieve a differential diagnosis with benign cardiac tumors [3, 8] and to show the extra cardiac extent and possible distant metastases [7]. Up to 80% of patients have distant metastasis at time of diagnosis [7].

Prognosis is correlated with several factors. In case of a complete surgical resection, life expectancy is twice as long as for an incomplete surgical resection [6, 7]. Another factor is necrosis, which is associated independently with poor survival. For non cardiac sarcomas histologic grading has been demonstrated to correlate with survival, but for cardiac sarcomas this could not be demonstrated. Tumors located in the left heart showed a better survival, which might be caused by an earlier discovery [6].

In the primary setting, surgical excision is the cornerstone of treatment and the value of about adjuvant chemotherapy or radiotherapy is not clear [1, 10]. No prospective studies are available to determine the efficacy of adjuvant (mostly anthracyclin based) chemotherapy and data are presented in single case reports [7, 11]. The same is the case for adjuvant radiotherapy. Fatima et al. retrospectively reviewed data and pathology slides of patients with primary angiosarcoma arising from the aorta, large vessels, and/or heart from 1975 to 2011. In total 13 patients were analyzed of which three patients had a angiosarcoma located in the heart. One patient underwent pericardiectomy, palliative chemotherapy and radiation therapy, about which no details were mentioned. This patient died 6 months from diagnosis [4]. In the cited case reports of patients with a primary cardiac sarcoma, patients received postoperative radiotherapy until a dose of 40 Gy to 54 Gy. Most of these patients had longer survival compared with surgery alone.

There are only a few case reports of patients who underwent postoperative radiotherapy, in which different schedules were used but all without severe toxicity. Pessotto et al. describe a patient with a recurrent primary cardiac leiomyosarcoma. At time of diagnosis the patient underwent surgery. When the tumor recurred 3 months later chemotherapy was administered which reduced the tumor mass almost to 10%. The patient received radiotherapy until a total dose of 40 Gy in 20 fractions. Forty months later the patient developed a local recurrence. The patient died eventually from brain metastases. This patient had the longest survival for primary cardiac leiomyosarcoma reported in the literature [8]. Another report describes a 47 year old patient with an angiosarcoma in the right atrium with adjuvant radiotherapy and chemotherapy after surgery. The patient was still alive 2 years after diagnosis [9].

Percy et al. describe a 22 year old patient with a primary angiosarcoma in the right atrium who received postoperative radiotherapy to the whole heart and mediastinum with the use of anterior and posterior ports. The port of radiation was reduced to the tumor site after the delivery of 39,6 Gy and an additional 10,8 Gy was delivered over 9 days. After the radiation therapy the patient received adjuvant chemotherapy which was discontinued by request of the patient. Thirty-four months after tumor resection patient was asymptomatic and free from disease [12].

In 1984 a case report was written about a patient with an angiosarcoma for which surgery was performed. Because the resection was probably irradical, postoperative radiotherapy was applied by two oblique anterior fields with wedge filters on most of the heart until a total dose of 50 Gy. Thirty-six Months after surgery the patient was in good health without signs of a local recurrence [13]. Another case report described a patient with an undifferentiated sarcoma in the right ventricle, pulmonary trunk and bilateral pulmonary arteries. The patient underwent surgery which was incomplete. Because the tumor recurred in several weeks chemotherapy was started without any effect on the tumor size and was stopped. External radiotherapy of the right ventricle and pulmonary arteries was performed until a total dose of 54 Gy in 30 fractions of 1,8 Gy. The patient died 6 months later of brain metastases [14].

Intimal sarcoma is the least frequent type of the primary cardiac sarcomas [2]. Three of our cases presented in this article had an intimal sarcoma. It is a poorly differentiated mesenchymal tumor and most often encountered in de large blood vessels and extremely rare in de heart [2]. To our knowledge, 9 cases are reported in the literature. Different treatment procedures were performed including surgery with or without adjuvant chemotherapy or radiation therapy [2, 15–17].

In the published case reports treatment plans were made using 3D conformal radiotherapy. With the current modern planning techniques, like Intensity Modulated Radiation Therapy (IMRT) and Volumetric Modulated Arc Therapy (VMAT), it is possible to reach a higher dose the tumor or the tumor bed with sparing of the organs at risk like the (not involved) heart and large vessels. Using a higher dose might lead to a better local control. In extremity sarcomas postoperative radiotherapy doses reach 60-70Gy to the original tumor location and 50Gy at the surgical bed. In some of the above mentioned reports doses of 40-54Gy were used, which is presumably too low for macroscopic disease.

The prognosis for patients with resectable cardiac sarcomas when treated with surgery alone is very poor with a mean survival of 3 months to 1 year [1]. Once metastasized, treatment options are palliative chemotherapy or targeted therapy which can be applied based on the histological sarcoma subtype with poor results [18, 19]. The patients who underwent resection followed by postoperative radiotherapy described in the above mentioned publicized case reports, all have a longer survival except one, which is also the case with our patients. We found a mean survival of 38 months (12–84 months). Six patients developed distant metastases of whom three also had a local recurrence. One patient had a local recurrence after surgery and adjuvant chemotherapy for which surgery and radiotherapy was performed and is still alive without signs of disease. Five patients had a survival of 2 years or more.

In conclusion, in this small case series of primary sarcomas of the heart, surgery combined with adjuvant radiotherapy is feasible in patients with resectable disease. In patients with an irresectable sarcoma of the heart primary radiotherapy should be considered.

## Data Availability

not applicable.

## Abbreviations

Gy: Gray
IMRT: Intensity modulated radiation therapy
VMAT: Volumetric modulated arc therapy
